# The developing and evaluation of an electronic tool to assess the effect of undergraduate training in palliative care: the electronic international medical education in palliative care (IMEP-e) assessment tool

**DOI:** 10.1186/s12904-019-0460-3

**Published:** 2019-09-02

**Authors:** Amrita Rai, Stephen Mason

**Affiliations:** 1 University of Liverpool, Liverpool, UK; 2 Palliative Care Institute Liverpool, Liverpool, UK

**Keywords:** Palliative care, Undergraduate education

## Abstract

**Background:**

With increased demand for palliative care (PC), the World Health Organisation (WHO) have called for PC teaching to be made routine. However, medical students report feeling unprepared in dealing with end-of-life care. Necessary benchmarking of the preparedness of clinicians to provide PC is required to identify where current training is sub-optimal and ensure future doctors are equipped to meet the needs of their patients. The aim of this study is to assess the utility of an electronic International Medical Education in Palliative Care (IMEP-e) assessment tool that examines the preparedness of clinicians to provide PC.

**Methods:**

A multi-phase pilot study.

*Phase 1:* To transpose the Self-Efficacy Palliative Care Scale (SEPCs) and the Thanatophobia Scale (TS) to an electronic format and evaluate its utility.

*Phase 2:* To assess the effects of PC teaching by comparing data from year three (Y3) and year five (Y5 - who have participated in PC placement) medical students.

Scales: *The 23 item SEPC* and *7 item TS* assess attitudes towards caring for dying patients.

**Results:**

Total questionnaires sent =360 (280 Y3, 80 Y5). Total response rate = 46.39%, *n* = 167 (127 Y3, 40 Y5). Completed data: *n* = 125 (95 Y3, 30 Y5). Analysis identified statistically significant differences (*p* < 0.001) between year groups across all subscales of the SEPC; communication skills (t = − 13.52), Pain and Treatment management (t = − 14.25) and multidisciplinary management (t = − 7.89). The TS shows a statistically significant increased positive attitudes (z = − 2.85 *p* < 0.005). From the focus group, three themes were identified from the qualitative feedback including university based teaching, hospice based teaching and utility of IMEP-e tool.

**Conclusion:**

The IMEP-e tool is a viable and comparable method for collecting data on the preparedness to practice PC. A larger scale study is needed to determine and evaluate if, and how, preparing clinicians to work in PC has been adapted in to routine training.

## Background

An increasingly ageing population [1] presents a global challenge for health care systems in how future care services are structured and managed, and how this impacts on patient welfare [2]. As the mean population age continues to rise, medical professionals of all grades and specialisations, will be required to provide palliative care (PC) for patients [3] with incurable chronic disease. These patients will increasingly present with multiple complex comorbidities and many will continue to die in hospital [4]. Physicians, irrespective of specialism or grade, should feel both competent and confident in caring for the dying patient [3].

The recent Lancet Commission report identified that half of all deaths in 2015 involved serious health-related suffering, equating to 6 billion hours of serious health related suffering across the globe each year [5]. To address these challenges, the World Health Organisation (WHO) have called for training in PC to be “integrated as a routine element” for all undergraduates in health related disciplines [6].

Within the UK the General Medical Council (GMC) have stipulated that learning to care for patients at end of life is compulsory in all undergraduate medical curricula [7]. However, little direct guidance is provided and as a result, Palliative and End of Life Care is under-prioritised at the discretion of the course co-ordinators [8]. Walker et al., (2016), has reported an increase in PC teaching delivered to undergraduates from 2000 to 2013 [9], but that PC teaching varied largely between medical schools, with not all students having direct clinical engagement. Clinical based teaching has been shown to be necessary in enabling medical students to learn how to effectively communicate, understand patients’ needs and perspectives, and appreciate the holistic approach required when planning clinical care [9]. Walker et al., (2016) concluded that PC teaching in the UK is ‘fragmented, ad hoc and lacking in coordination and consistency’ [9].

Although all medical schools in the UK provide some form of training, recent reports from the British Medical Association (BMA) have pleaded that better education and training in end-of-life issues should be available [10]. Although many medical students practice with simulated patients, often the first contact with PC patients is after qualification [3]. Accordingly, junior doctors report that PC is an area in which they feel most “unprepared and which causes them the greatest distress,” [11]. This is present still, as the current data shows confidence levels remain low amongst doctors at all levels [3, 10]., For example, in a recent study which interviewed Foundation Year 1 doctors (first year following qualification), from different medical schools, the doctors reported feeling insufficient time was dedicated to PC within the undergraduate degree, leaving doctors unprepared to deal with death and the dying on wards [12].

### Assessment of undergraduate training in palliative care

The Palliative Care Institute Liverpool has developed a theoretically underpinned, psychometrically tested assessment tool (the *Self Efficacy in PC scale (SEPCs)),* and used this in parallel with the *Thanatophobia* scale (TS), to provide a single numerical indication of how prepared a medical student or newly qualified doctor is when facing PC situations [13]. To date, these assessment tools have been used in over 13 countries worldwide and translated into 6 languages.

The assessment tools have been used in a large international study [14], and have recently been converted into a multi-platform accessible electronic format: the **I**nternational **M**edical **E**ducation in **P**alliative Care - **e**lectronic tool (IMEP-e). The aim of this study is to pilot the electronic tool with undergraduate medical students at a University Medical School in the North of England. This is because the tool has never been used in an online format before (only paper). Piloting the electronic format is important to assess the feasibility of using this tool in assessing PC preparedness, including factors such as response rate and completeness of the questionnaire. A secondary aim was to examine if the IMEP-e would be sensitive enough to identify a hypothesised difference in preparedness between the medical students who were yet to receive palliative care training (3rd year students) and those who had (5th year students).

## Methods

### Design

A mixed methods approach was undertaken to assess the utility and user-friendliness of the IMEP-e tool’s potential to record meaningful data.

### Sample

Convenience sampling was used with undergraduate medical students (Y5 and Y3) at a University in the North West of England. The two different cohorts were used as Y5 has received training in PC and Y3 had not. A smaller Y5 sample group is used due to the wider distribution of the students in clinical placement, as we only recruited students from the two base hospitals.

### Procedure

In the academic year of 2017, Y3 and Y5 student doctors were sent a one off email inviting them to participate in the study, providing them with a study/ participant information sheet and a weblink to complete the IMEP-e tool. Y5 students that responded positively to the IMEP-e tool were sent an invitation to attend a focus group. The aim of the focus group was to:

1. Assess the utility of the IMEP-e tool.
2. Assess the impact of PC undergraduate training.

The focus group was audio recorded, and the recordings transcribed to enable qualitative analysis.

### Measures

The IMEP-e tool consists of:

1. *Demographic Information*: including details on hours of training in palliative care and perceived support during training.
2. *Self-Efficacy Palliative Care Scale (SEPCs)* [13]: a 23 item self-assessment questionnaire that queries how clinicians manage various situations in a PC environment e.g. ‘discussing issues of death and dying’. The SEPC has three distinct subscales focussing on; Communication Skills (Comm); Multidisciplinary teams (MDT) and Pain and Symptom Management (PSM). Possible answers ranged from 0 (very anxious) to 100 (very confident) on a visual analogue scale. For example Q1 “discussing the likely effects of cancer with a patient” – a score of 20/100 would indicate the student is anxious about this situation.
3. *Thanatophobia scale (TS)* [15]: a 7-point Likert scale with 7 questions examining the expected personal outcomes of a health care professional across a range of clinical situations in caring for a dying patient. Possible answers ranged from 1 (strongly disagree) to 7 (strongly agree). For example Q25 “managing dying patients traumatises me” – a score of 2/7 would indicate the student disagrees with this statement.

### Analysis

Descriptive statistics were used to examine participation and return rates. T-test and Mann-Whitney U were used to examine the difference in clinical preparedness between Y3 and Y5, with inferential analysis applied on all SEPC subscales and TS: statistical analysis was performed using the software Statistical Package for the Social Sciences (SPSS) [16]. Qualitative thematic analysis using Braun and Clarke’s approach [17] was used to examine the data generated from the focus group. The focus group recording was transcribed and divided into common themes. Within these themes, subthemes were identified. These themes and subthemes contain the quotes from the students that most reflect the focus group discussions.

Ethical approval in March 2018 from the University of Liverpool, Health and Life Sciences Committee was granted for this study: reference number: 1435. Electronic written consent was obtained from all participants taking place in the study, with all participants ticking a consent box.

## Results

**Fig. 1:**
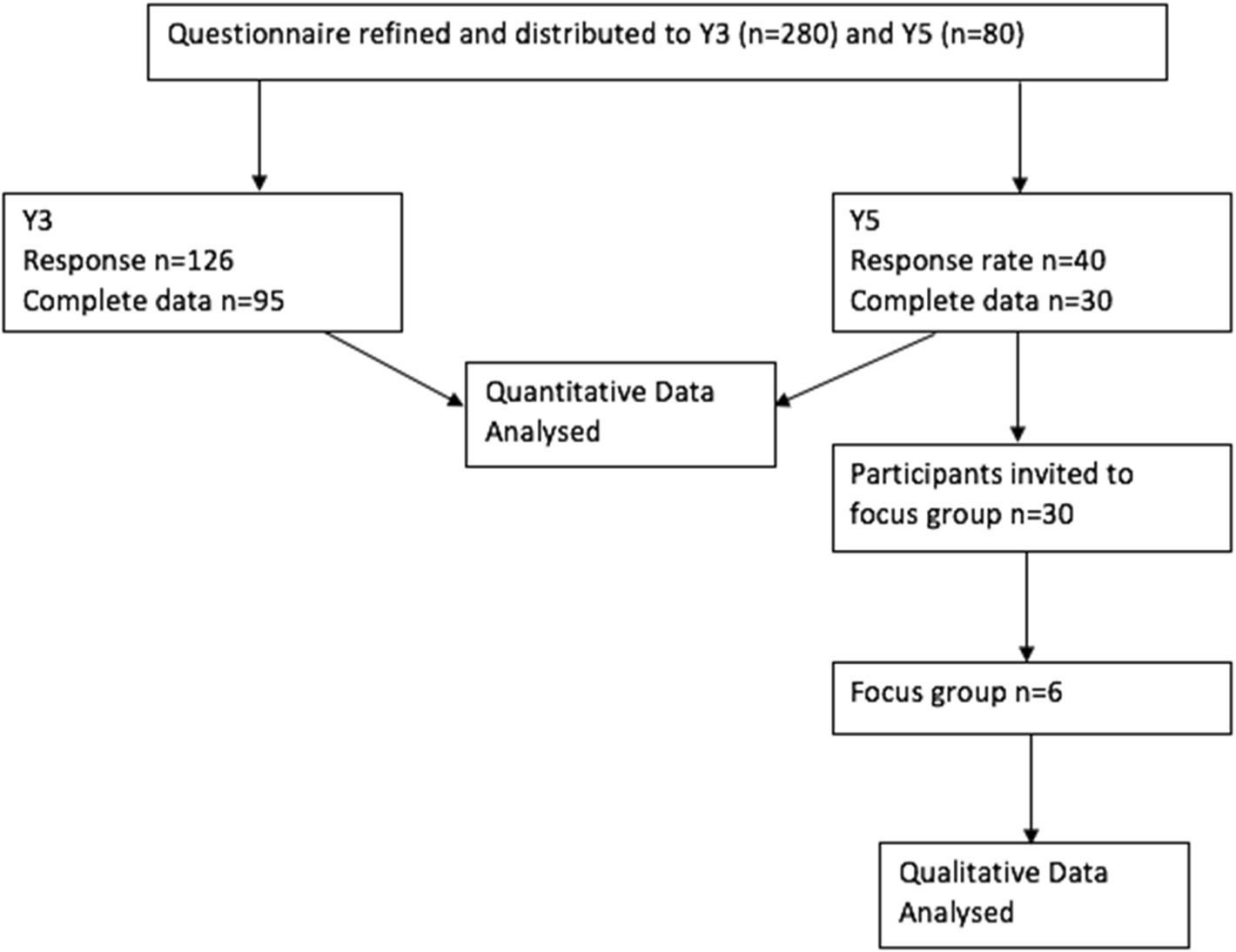
Flowchart to summarise methodology

In total, 125/360 (35%) medical students participated. By year of study, 95/280 (34%) of Y3 students and 30/80 (37.5%) of Y5 students completed the study. Figure 1 details the recruitment process.

There are large differences in mean scores on the SEPC subscales, which when examined using an independent t-test, shows a statistically significant difference between the groups (Table 1). For example, on the subscale communication (Comm), students in year 3 reported significantly lower confidence (SEPC = 23.51) than those in year 5 (SEPC = 47.50, t = 13.52, *p* = 0.001). Further analysis using the data provided also indicates a large effect size, which is portrayed as a high Percentage (e.g. 70%) of Non-Overlap, indicating an educationally meaningful difference between the cohorts, in addition to statistical difference.

**Table 1:** T-values of SEPC and effect size

Scale	Mean Scores		*t*-value	*p*	Cohen’s *d*	Effect Size	Percentage Non-Overlap
	Y3	Y5					
SEPC - Comm.	23.51	47.53	−13.52	.001	−1.57	Large	> 70.7%
SEPC - Pt Man.	28.71	54.98	−14.25	.001	−1.98	Large	> 79.4%
SEPC – MDT-w/ing	29.12	54.01	−7.89	.001	−1.73	Large	> 75.4%

Table 2 reflects the attitudes towards caring for PC patients from questions 24–30 in the questionnaire, which use the TS. Analysis for statistical difference between each of the items, and the total score of the TS show a statistically significant difference overall (Thana), despite both groups expressing positive attitudes in their views of caring for dying patients. The smallest Z number was − 3.99 (Q25 - *I feel pretty helpless when I have terminal patients on my ward*), which indicates less variation of opinion between the two respective cohorts. The largest Z number was − 1.58 (Q29 - *I don’t look forward to being the personal physician of a dying patient*), showing less variety in opinion across the two cohorts.

**Table 2:** Analysis of Thanatophobia Scale (TS)

	Q24	Q25	Q26	Q27	Q28	Q29	Q30	Thana
Mann-Whit. U	997.5	743.5	1149.0	998.5	1131.0	1156.5	1088.0	933.0
Z-score	−2.53	−3.99	− 1.63	−2.51	− 1.73	− 1.58	−1.98	−2.85
Sig. (2-tail)	0.01	0.000	0.10	0.01	0.08	0.11	0.05	0.01

Despite both groups expressing positive attitudes in their views for caring for dying patients, a statistically significant difference in attitude was observed, favouring Y5 cohorts.

### Qualitative data – focus group

Three core themes and a number of sub-themes were identified from the focus group discussion. The core themes include: *University based teaching, Hospice based teaching* and *Utility of IMEP-e.*

*University based teaching* includes the subthemes: Classroom and Simulation teaching, Broad curriculum information and Lecture content. Communication training, which was included in classroom and simulation training, was identified as the most valued aspect of the module in the qualitative data.

*“They have really good workshops for discussing spiritual care and communication skills.” Participant 1.*

*“The good thing about communication sessions... is that you get feedback on how you’re speaking to people.” Participant 2.*

Some students thought that the PC module in fourth year should be integrated and spread throughout the clinical years, rather than focused into a single module in fourth year.

*“We got quite a lot of new information, because we hadn’t done palliative care (before), we didn’t know much about it.” Participant 3.*

Y5 students highlighted that more PC education throughout training would be beneficial, especially as they have exposure to PC patients in clinical scenarios without specific PC training;

*“You start being in specialities in third year. I don’t think there would necessarily be anything wrong with bringing a bit more palliative into third year as well.” Participant 5.*

#### *Hospice based teaching* included the subthemes

Clinical and bedside teaching in hospice, Practical skills preparation for F1 role, Patient focused holistic needs and Individual student support.

The theme received positive feedback about aspects of the module such as needs of a PC patient.

*“I want to work in primary care and I feel as though a lot of home visits will be with a lot of terminally ill patients, and I think I have gotten a greater understanding about medications and the holistic care they need.” Participant 1.*

*“I’ve definitely got a better idea of the management and the actual medical role as well as the emotional role in breaking bad news and the management of the patient.” Participant 5.*

It was noted that there was a range of support that was given to the students to support their learning, and also, to assist if the students were also experiencing personal challenges; such as a chronically ill family member or recent bereavement.

*“…we (medical students) were contacted beforehand as well, you know, if there was anything that you might be going through.” Participant 4.*

*“…they did sort of give us an interview at the start of the placement on the first day. Just to see what you would want to be getting out of the placement and anything they should be initially aware of.” Participant 4.*

The qualitative research particularly highlights the student satisfaction in a student centred approach to teaching, with the students expressing the benefits in terms of psychological and professional support when faced with dying patients, especially when some students became emotional during the module;

*“The staff were amazing. They talked to them (medical students), took them to a side room, and for an hour they were really, really supportive.” Participant 3.*

#### *Utility of IMEP-e* included the subthemes

Online accessibility, Student evaluation of questionnaire, Interpretation of questionnaire subscales and Analysis of SEPCs and TS used.

There was positive feedback on the utility of the questionnaire, however some effects of repetition were noted.

*“The questionnaire was really useful.” Participant 3.*

*“I thought all the questions were relevant.” Participant 5.*

*“My answers became a little bit oversaturated, maybe. Especially on the analogue scale, I felt like some of them were quite similar.” Participant 7.*

There was discussion about various issues when accessing the IMEP-e. It was noted that some students had problems initially accessing the site. Overall, there was very positive feedback with the wording and content of the questionnaires.

*“There were some problems logging on, it didn’t work on Safari.” Participant 2.*

*“It wasn’t too difficult the questions as well, they were understandable.” Participant 6.*

The formatting and user-friendliness of the IMEP-e was commented on, with students preferring the visual analogue scale options to record their perceived efficacy.

*“It was good when you could set between the bar.” Participant 6.*

The Y5 focus group provided rich qualitative data on both the utility of the IMEP-e and the experience of learning the practice of PC. The IMEP-e was reported as useful and relevant.

Elicited from all themes was the students’ overall appreciation for the PC module. The students did not consider PC as a ‘soft’ subject but a subject that needs more emphasis, as these students do not want to be placed in clinical situations without the requisite skills to support patient care.

## Discussion

The results presented and the comments from the focus group, suggest that the IMEP-e is appropriate in its use, easily understood and is a suitable reflection of PC teaching in the undergraduate curriculum. Analysis of the findings showed that the Y5 cohort are significantly more confident and prepared for PC compared with the Y3 cohort.

### Utility of IMEP-e tool

The focus group gave rich qualitative data about the utility of the IMEP-e, claiming that it’s “useful”, “relevant”, and “user-friendly”. In line with the pilot nature of the study there were technical challenges identified that were discussed in the focus group, such as problems logging on and using the unique access code, along with some pages in the questionnaire being unable to load. This has been attributed to differences in software and Internet Server Platforms, as subsequent examination has identified challenges with certain providers such as Safari. As a pilot study, these issues will need to be resolved before further application.

### Quantitative data

Analysis of the questionnaire data overall suggests that the 4th Year PC module actively prepares medical students for practice and improves attitudes towards care (Tables 1 and 2). Furthermore, the effect size calculations are helpful in identifying indicating a clear and meaningful difference in the preparedness between both groups, above the statistical significance reported. This indicates that on completing the PC rotation, Y5 medical students felt much more prepared to take on a responsible role in PC. This may also reflect general clinical experience and exposure as they are closer to their F1 role than the Y3 cohort.

Both year groups had positive attitudes towards caring for dying patients (TS < 4), with Y5 attitudes being significantly more positive. The most significant difference between the 2 year groups was in Q.25; “*I feel pretty helpless when I have terminal patients on my ward.*” This could be because Y5 students felt more capable of asking for help and knowing practical responses to difficult situations after the PC module. Some anomalies were identified in the analysis of the individual items within the TS; where no statistical differences between Y3 and Y5 were recorded. For example, Q.28 “*It makes me uncomfortable when a dying patient wants to say goodbye to me,”* and Q.29, “*I don’t look forward to being the personal physician of a dying patient.*” This may be because the module did not change their opinion to these questions; or this could be attributed to the beliefs people have as individuals; or that the attitudes in the students before the module was already positive.

### Qualitative data

The focus group conducted by Y5 students supplied qualitative data regarding the students experience of the PC module and the utility of the IMEP-e tool. The most valued aspect of the module was communication training, this is particularly interesting as medical students typically have less communication training later in the degree, however, in the response to this focus group, perhaps the students would appreciate more communication training in the form of simulated patients and ward based communication skills. This is also reflected by the quantitative data collected, where Y5 students expressed more confidence in Comm. skills post-PC module.

The focus group also drew prominence to the request for earlier exposure to the dying patient. This may have benefits as there is a lot of exposure to PC patients in all hospital settings, as Clark reports that “almost 1 in 10 patients in teaching or general hospitals at any given time will die during that admission” [18]. Without appropriate training, students may find themselves disadvantaged in such a setting;

*“We get clinical placement from second year without really having that kind of experience until fourth year, so there’s a lot of time to make some silly comments that we could have avoided had we integrated it a bit earlier, it would have helped us in hospital a bit as well.” Participant 4.*

In addition, the integration of PC educational teaching earlier in the curriculum may reduce the burden of information in fourth year. This was flagged in the focus group as some felt overwhelmed with fourth year PC content;

*“in a way the module was trying to overload you with everything in that one rotation in fourth year…”. Participant 4.*

However, if the information is made more manageable over two years instead of one, this may be easier to comprehend and retain.

### Strengths and limitations of the study

The study provided encouraging results for the success of the PC module, showing statistical and educationally significant improvements after the PC training. The qualitative data portrayed the utility and relevance of PC teaching. Interesting findings were also elicited, such as the appreciation for student support when facing dying patients. Students were open about their emotional vulnerability; it is therefore valuable that this was addressed in a safe, undergraduate environment with ample support, compared to a potentially more hostile one when newly qualified doctors may be facing these patients alone for the first time.

The piloting of an electronic questionnaire from an original paper copy is also advantageous for economic, environmental and convenience purposes.

The response rate with complete data from the IMEP-e was 34% from Y3 students and 37.5% for Y5 students. Although the response rate from an opportunity sample can be low [19], there is discrepancy between the number of students that started to respond to the IMEP-e and those that had complete the data. The focus group described mostly technical difficulties, and therefore, on solving these issues, the response rate in the future should be higher. If the response rate is still low in future, there may be further improvements required.

Medical students are also a specific group who are fatigued by the many surveys they are expected to complete. Considering this, the response rate of this group may be a reflection of the questionnaire’s ease of accessibility, registration and use. This online format can be seen as more preferential compared to other types of questionnaire such as postal.

#### Female dominated response

There is a much larger response from females compared to males (about 1:4) shown in Table 3. This may reflect the changing female dominance in the medical profession, and the gender shift in medicine as more women engage with the medical degrees compared to men [20]. Although most of the staff overall in PC are female, there are more male consultants. The qualitative data was collected in the form of a focus group. The sample consisted of six male participants, and this may have impacted the results collected. It is possible to suggest that males may have a different outlook on sensitive issues such as PC in comparison to females.

**Table 3:** Demographics of participants

	3rd Years	5th Years	Focus group
Male: Female	17:78	16:14	6:0
Age range (years)	21–27	22–31	22–25
Median Age	22	23	24

### Future research

With the IMEP-e developed and amenable to administration, future research will involve following medical students throughout the current undergraduate curriculum and subsequent foundation training to assess the effect of current medical training. Gibbons et al. in their 2011 audit suggest that few medical students were aware they would be directly caring for patients with incurable progressive conditions [6]. As previously stated, the WHO require PC to be integrated in the undergraduate curriculum [6]. However, for a meaningful change in preparedness felt by junior doctors [11], and newly qualified foundation doctors [12], an effective baseline measurement needs to be assessed, preferably conducted at an undergraduate level nationally across medical schools. This may be the future direction of this pilot study, as the data utilised in this study was collected from a cohort representative of just one medical school.

The undergraduate curricula vary between institutions, therefore national data would provide important information on the effects of individual medical school training and provide guidance on where to improve the competencies of future doctors and more importantly, reduce the challenges faced by current foundation doctors who are caring for PC patients. Similar work can also be undertaken internationally, utilising the currently available translation of the original tools in German, French, Italian, Spanish, Brazilian Portuguese and Mandarin.

## Conclusion

The results from this study indicated that the IMEP-e is an effective and efficient way to assess the effects of undergraduate training in PC, and that considered and structured education appropriately prepares student doctors for practice.

The electronic platform and database created in the development of the IMEP-e will enable potentially large and longitudinal assessments of the effect of undergraduate training, equipping medical schools and providing student doctors with the training required to meet the needs of the patients they will care for. Furthermore, the multiple translations of the IMEP-e will also facilitate national and international comparisons, which in turn may promote collaborative learning and drive the development and adoption of best practice in preparing tomorrow’s doctors.

## Data Availability

The datasets used and/or analysed during the current study are available from the corresponding author on reasonable request.

## Abbreviations

AR: Amrita Rai (author)
BMA: British Medical Association
BMC: British Medical Council
Comm: Communication skills
F1: Foundation Year 1 (doctors)
GMC: General Medical Council
IMEP-e: Electronic International Medical Education in Palliative Care (assessment tool)
MDT: Multidisciplinary teams
PC: Palliative care
Post-PC module: Post palliative care module
PSM: Pain and symptom management
Q1: Question 1
SEPC: Self-efficacy palliative care (scale)
SM: Stephen Mason (author)
SPSS: Statistical Package for the Social Sciences
TS: Thanatophobia scale
UK: United Kingdom
WHO: World Health Organisation
Y3: Year 3 (medical students)
Y5: Year 5 (medical students)
